# Epidemiology and Clinical Outcomes of Microscopic Colitis: Preliminary Results From the Loyola University Microscopic Colitis Registry (LUMiCoR)

**DOI:** 10.3389/fmed.2021.715458

**Published:** 2021-09-20

**Authors:** Poornima Oruganti, Rehmat Awan, Xianzhong Ding, Michael Wesolowski, Ayokunle T. Abegunde

**Affiliations:** ^1^Department of Internal Medicine, Loyola University Medical Center, Maywood, IL, United States; ^2^Department of Pathology, Loyola University Medical Center, Maywood, IL, United States; ^3^Loyola University Chicago, Clinical Research Office Biostatistics Collaborative Core, Chicago, IL, United States; ^4^Division of Gastroenterology and Nutrition, Loyola University Medical Center, Maywood, IL, United States

**Keywords:** microscopic, colitis, clinical, epidemiology - descriptive, outcomes

## Abstract

Microscopic colitis (MC) is a common cause of chronic diarrhea with limited long-term data. We searched the pathology records at our institution from 2008 to 2018 to identify cases of MC. Total sample included patients with either a diagnosis of MC or incomplete MC (MCi).Chart review was performed and data were summarized for descriptive statistics. Logistic regression was used to estimate the unadjusted effects of predictors on MC. A total of 216 patients (88.32% white, 80.56% females, mean age 67.12 +/– 15.79) were studied; 50.00% had CC, 40.28% had LC and 9.72% had MCi. Majority (52.31%) were smokers and 21.84% of females were using some form of hormonal therapy. The odds of LC in reference to CC were significantly higher for those using tricyclic antidepressants (TCAs) (OR: 3.23, 95% C.I: 1.18–8.80, *p* = 0.02). The odds of smoking, statins, aspirin and beta-blocker use were decreased in MCi in reference to CC (all *p* < 0.05), 29 (74.35%) patients with unresolved symptoms underwent repeat colonoscopies with biopsies. One case of MCi resolved, 8 (72.73%) out of 11 cases of LC resolved, 2 (18.18%) continued to be LC and 1 (9.09%) transformed to CC, 8 (47.06%) out of 17 cases of CC resolved, 8 (47.06%) continued to be CC and 1 (5.88%) transformed to LC. Majority of patients had CC. TCA use resulted in increased odds of LC in reference to CC. Biopsies from repeat colonoscopies in some patients revealed changes in the pathological diagnoses raising the question of interchangeability of MC (CC to LC and vice versa).

## Introduction

Microscopic colitis (MC) is a common cause of chronic, watery diarrhea with limited long-term data. MC is used as an umbrella term to categorize a subgroup of colitides with distinct clinicopathological phenotypes and no significant endoscopic abnormalities (1). The prevalence of MC exceeds 20 per 10^6^ in many countries (1–5) and MC is found in approximately 10–15% of patients with chronic watery diarrhea undergoing colonoscopy with biopsy, with higher detection rates in the elderly and females (6). MC consists of 2 distinct histopathological diagnoses: Lymphocytic colitis (LC) marked by >20 intraepithelial lymphocytes (IELs) per 100 epithelial cells (ECs), and Collagenous colitis (CC) characterized by > or <20 IELs/100 ECs and a thickened subepithelial collagen band (>10 microns). Variant forms with fewer characteristic features have been reported: Incomplete Collagenous Colitis (CCi) and Incomplete Lymphocytic Colitis (LCi). CCi or LCi may represent different manifestations during the disease course or different stages of disease development (7). Substantial clinical and histological overlap between LC and CC has been described, raising the suspicion that both conditions are two histological manifestations of the same entity, possibly representing different manifestations during the disease course or different stages of disease development (1). We aim to describe the local epidemiology and risk factors for MC and describe histological changes in a sub-group of patients with MC who underwent follow up colonoscopy after treatment.

## Methods

We conducted a natural language search of the pathology records at our institution from 2008 to 2018 using the words “lymphocytic colitis” and “collagenous colitis.” The total sample included patients with either a diagnosis of MC or MCi (CCi/LCi). Histological descriptions were based on hematoxylin and eosin (HE) staining. Special stains were applied in borderline cases: immunohistochemical staining for CD3 and Trichrome stain were used to detect LC and CC respectively. We used the original pathology reports as these were performed by expert pathologists with knowledge of MC during the study period. The electronic medical record (EMR) of each patient was reviewed to obtain data on demographics, clinical symptoms, comorbidities, medication, diagnosis, treatment, and resolution of symptoms. The study was approved by the Institutional Review Board of Loyola University Medical Center, Maywood, Illinois.

### Statistical Analysis

Descriptive summary statistics are reported as frequencies and percentages for categorical variables, and as means and standard deviations for numerical variables. Frequencies and column percentages are reported to describe the bivariate relationships between categorical predictors and type of microscopic colitis diagnosis. Stratified medians and interquartile ranges are reported for numerical predictors. Univariable multinomial logistic regression models are used to estimate the effects of predictors on the odds of incomplete colitis MCi (CCi/LCi), and on the odds of lymphocytic colitis (LC), in reference to collagenous colitis (CC). Wald 95% confidence intervals and Chi square *p*-values are reported for each odds ratio estimate. Frequencies and row percentages are reported to describe the bivariate associations between monotherapy and symptom resolution, and between combination therapy and symptom resolution. Fisher's exact test was used to evaluate the association between combination treatment therapies and symptom resolution.

## Results

A total of 216 patients (88.32% white, 80.56% females) with mean age 67.12 +/– 15.79 were studied; 50.00% had CC, 40.28% had LC and 9.72% had MCi. Majority (52.31%) were smokers and 21.84% of females were using some form of hormonal therapy (Table 1). The odds of LC in reference to CC were significantly higher for those using tricyclic antidepressants (TCAs) (OR: 3.23, 95% CI: 1.18–8.80, *p* = 0.02; Table 2). The odds of smoking, and use of statins, aspirin, and beta-blockers are decreased in MCi in reference to CC (all *p* < 0.05; Table 3). A total of 166 patients had symptom resolution data. Majority (127 of 166, 76.50%) had resolved symptoms. Patients received interventions such as stoppage of offending medications, dietary modification, and/ or a variety of treatments such as antidiarrheals, bismuth subsalicylate, 5-Aminosalicylates, prednisone, budesonide, bile-acid binders, anti-TNF agents, azathioprine, methotrexate, antibiotics, fiber, and surgery. Treatments were grouped into monotherapy (exclusively one of the aforementioned treatments) and combination therapy (any combination of treatments). Combination therapy was divided into the following subgroups: budesonide combination therapy (budesonide + any of the aforementioned treatments), non-budesonide combination therapy (combination of any of the aforementioned treatments excluding budesonide), and other (immunomodulators, anti-TNF, antibiotics, surgery). The association between monotherapy and symptom resolution could not be evaluated with a statistical hypothesis test, due to sparse, insufficient data (Appendix 1). There was no significant association between combination therapy and symptom resolution (*p* = 0.37; Appendix 2). Twenty-nine (74.35%) patients with unresolved symptoms underwent repeat colonoscopies with biopsies. One case of MCi resolved. 8(72.73%) out of 11 cases of LC resolved, 2(18.18%) continued to be LC and 1(9.09%) transformed to CC. 8(47.06%) out of 17 cases of CC resolved, 8(47.06%) continued to be CC and 1(5.88%) transformed to LC.

**Table 1:** Clinical characteristics of patients.

**Variable**	***n***	**Summary measure**
**Age** (years), Mean (SD)	216	67 (16)
**BMI**, Mean (SD)	215	27.2 (6.2)
**Sex**, n (%)	216	
Female		174 (80.6)
Male		42 (19.4)
**Race** n (%)	214	
Non-white		25 (11.7)
White		189 (88.3)
**Hormonal Therapy**	174	
Yes		38 (21.8)
No		136 (78.2)
**Smoking**, *n* (%)	216	
Yes		113 (52.3)
No		103 (47.7)
**Diagnosis**	216	
Collagenous colitis (CC)		108(50.0)
Lymphocytic colitis (LC)		87(40.3)
Microscopic colitis “incomplete” (MCi)		21(9.7)

**Table 2:** Association of smoking and medication with LC compared with CC.

**Variables**	**LC *n* = 87**	**CC (REF)*n* = 108**	LC vs. CC OR (95% CI)^*^	***p*-value**
**Smoking**				
Yes	44 (50.6)	62 (57.4)	0.76(0.43, 1.34)	0.34
No	43 (49.4)	46 (42.6)		
**Statins**				
Yes	48 (55.2)	63 (58.3)	0.88(0.50, 1.55)	0.65
No	39 (44.8)	45 (41.7)		
**Tricyclic Antidepressants**				
Yes	14 (16.1)	6 (5.6)	3.23(1.18, 8.80)	0.02^*^
No	73 (83.9)	101(94.4)		
**Aspirin**				
Yes	40 (46.5)	64 (59.3)	0.60(0.34, 1.06)	0.07
No	46 (53.5)	44 (40.7)		
**Beta Blocker**				
Yes	33 (37.9)	47 (44.3)	0.77(0.43, 1.37)	0.89
No	54 (62.1)	59 (55.7)		

* *Logistic regression; unadjusted odds ratio with 95% confidence interval (C.I.). P-value < 0.05 statistically significant*.

**Table 3:** Association of smoking and medication with MCi compared with CC.

**Variable**	**CCi/LCi *n* = 21**	**CC (REF)*n* = 108**	CCi/LCi vs. CC ^*^ OR (95% CI)	***p*-value**
**Smoking**				
Yes	7 (33.3)	62 (57.4)	0.37(0.14, 0.99)	0.04
No	14 (66.7)	46 (42.6)		
**Statins**				
Yes	5 (23.8)	63 (58.3)	0.22(0.08, 0.65)	0.006
No	16 (76.2)	45 (41.7)		
**Tricyclic Antidepressants**				
Yes	1 (4.8)	6 (5.6)	0.84(0.10, 7.38)	0.87
No	20 (95.2)	101 (94.4)		
**Aspirin**				
Yes	5 (23.8)	64 (59.3)	0.22(0.07, 0.63)	0.005
No	16 (76.2)	44 (40.7)		
**Beta Blocker**				
Yes	3 (14.3)	47 (44.3)	0.21(0.06, 0.75)	0.017
No	18 (85.7)	59 (55.7)		

* *Logistic regression; unadjusted odds ratio with 95% confidence interval (C.I.) P-value < 0.05 statistically significant*.

## Discussion

The etiology of MC is unknown, several risk factors such as smoking, use of Non-steroidal anti-inflammatory drugs (NSAIDs), proton pump inhibitors (PPI), antidepressants, statins, and hormonal therapy have been reported, but no causal association has been established (1–3, 8). Consistent with previous studies, this study shows that MC predominantly affects females, and the mean age at diagnosis is 67+/−16 years, suggesting that our study population is adequately representative of the population of MC patients.

Similar to previous studies (2) the majority of patients in our cohort had CC. The odds of LC in reference to CC were significantly higher for those using tricyclic antidepressants (TCAs), a new finding that has not been previously reported. TCAs are commonly prescribed for patients with functional GI disorders and the prevalence of MC in patients with IBS-D is approximately 10%. Therefore, that finding is important and deserves further study. Patients with MCi had reduced odds of smoking, and use of statins, aspirin, and beta-blockers compared with patients with CC. Previous studies revealed that histologic findings in CC persist for a longer period compared with LC or MCi, (8, 9) therefore CC was used as the reference diagnosis for comparison.

Many patients with symptomatic MC do not suffer long term symptomatic disease if they achieve clinical remission. Evidence from the published literature suggests that stoppage or avoidance of the aforementioned risk factors in symptomatic MC patients can result in improvement or resolution of symptoms in a sub-set of patients (8).

However, majority of MC patients require specific treatment with antidiarrheals or Budesonide (8, 9). The estimated rate of clinical and histological improvement in patients with symptomatic MC treated with budesonide is approximately 81% (9). However, despite effective treatment, approximately 30–60% of MC patients may relapse (10, 11). Therefore, relapse or persistent symptoms can lead to poor quality of life and increased health care costs (8). In most cases, reinitiating Budesonide treatment leads to clinical remission, which can be maintained with a lower dose (8, 9).

This study shows that 76.50% of patients had resolved symptoms, while 23.50% had persistent or recurrent diarrhea. The association between different treatments and symptom resolution was assessed by comparing treatments in patients with resolved and unresolved symptoms. Due to insufficient data the association between monotherapy and symptom resolution could not be assessed. There was no significant difference in symptom resolution between patients that received budesonide combination therapy and non-budesonide combination therapy (*p* = 0.37).

In a sub-group of patients in our cohort with persistent or recurrent diarrhea, biopsies from repeat colonoscopies revealed changes in the pathological diagnosis raising the question of interchangeability of MC (CC to LC and vice versa). Rasmussen et al. reported similar findings and showed that MC persisted for up to one year in 77% with CC, 64% with LC and 45% with MCi, of whom 6, 9 and 18% respectively changed to a different MC subgroup (12). A possible explanation for interchangeability of diagnosis is that CC can have a patchy distribution of collagen throughout the colon and biopsies may show only intraepithelial lymphocytosis, consequently the biopsies may be interpreted as LC (13). Another explanation could be suboptimal orientation of biopsies which sometimes hinders assignment to a definitive MC subgroup (12).

Limitations of our study include the retrospective single center design and relatively small number of patients with repeat colonoscopy and biopsy. Non-randomized allocation of treatments and incomplete data on symptom resolution. Therefore, our results need to be prospectively validated in a larger population. Future prospective studies are needed to clarify the natural history, identify novel risk factors, and track outcomes of patients with MC using validated measures of disease activity.

## Data Availability Statement

The raw data supporting the conclusions of this article will be made available by the authors, without undue reservation.

## Ethics Statement

The studies involving human participants were reviewed and approved by Loyola University Chicago Institutional Review Board. Written informed consent for participation was not required for this study in accordance with the national legislation and the institutional requirements.

## Author Contributions

AA planning and conducting the study, collecting and interpreting data, and revising the manuscript. PO and RA collecting and interpreting data and drafting the manuscript. XD collecting and interpreting data and revising the manuscript. MW planning the study, statistical analysis, and revising the manuscript. All authors contributed to the article and approved the submitted version.

## Conflict of Interest

The authors declare that the research was conducted in the absence of any commercial or financial relationships that could be construed as a potential conflict of interest.

## Publisher's Note

All claims expressed in this article are solely those of the authors and do not necessarily represent those of their affiliated organizations, or those of the publisher, the editors and the reviewers. Any product that may be evaluated in this article, or claim that may be made by its manufacturer, is not guaranteed or endorsed by the publisher.
