# Supplementary material for: Epidemiology and Clinical Outcomes of Microscopic Colitis: Preliminary Results From the Loyola University Microscopic Colitis Registry (LUMiCoR)
Source: Front Med (Lausanne). 2021 Sep 20;8:715458. doi: 10.3389/fmed.2021.715458 (PMC8493955; doi:10.3389/fmed.2021.715458)
Supplement: Supplementary file 1 [file Data_Sheet_1.pdf]

Appendix 1: Bivariate Association of Monotherapy & Symptom Resolution

Monotherapy	Symptom Resolution	
	Unresolved Symptoms	Resolved Symptoms
Bile Acid Binder	0 (0.00)	1 (100.00)
5-Aminosalicylates	0 (0.00)	5 (100.00)
Bismuth Subsalicylate	2 (28.57)	5 (71.43)
Steroids (Budesonide or Prednisone)	13 (20.97)	49 (79.03)
Antimotility Agent	2 (25.00)	6 (75.00)
Fiber	1 (25.00)	3 (75.00)
Dietary Changes / No Specific Treatment / Stopping or Avoiding Offending Medication	2 (13.33)	13 (86.67)

Appendix 2: Bivariate Association of Combination Therapy & Symptom Resolution

Combination Therapy	Symptom Resolution	
	Unresolved Symptoms	Resolved Symptoms
Other Treatments*	3 (60.00)	2 (40.00)
Non-Budesonide Combination Therapy	4 (26.67)	11 (73.33)
Budesonide Combination Therapy	12 (27.27)	32 (72.73)

*Other treatments: Anti-TNF, Immunomodulators, antibiotics , surgery
Budesonide combination therapy= Budesonide + any of the treatments listed in appendix 1
Non-Budesonide therapy= Any combination of treatments without budesonide
